# Soluble CSF1R alleviates microgliopathy in a CSF1R-related leukoencephalopathy (CRL) mouse model

**DOI:** 10.1186/s12974-025-03648-4

**Published:** 2025-12-05

**Authors:** Yuhang Zhou, Banglian Hu, Jie Luo, Xingyi Wang, Xiaohua Huang, Yanfang Li, Xian Zhang, Guojun Bu, Hongsheng Zhang, Yun-Wu Zhang, Honghua Zheng

**Affiliations:** 1 Xiamen Key Laboratory of Brain Center, The First Affiliated Hospital of Xiamen University, Fujian Provincial Key Laboratory of Neurodegenerative Disease and Aging Research, Institute of Neuroscience, School of Medicine, Xiamen University, Xiamen, Fujian 361102 China; 2 Basic Medical Sciences, School of Medicine, Xiamen University, Xiamen, Fujian 361102 China; 3 Division of Life Science and State Key Laboratory of Nervous System Disorders, The Hong Kong University of Science and Technology, Clear Water Bay, Hong Kong, China; 4 Department of Neurobiology, School of Basic Medical Sciences, Key Laboratory of Major Brain Disease and Aging Research (Ministry of Education); Department of Neurology, The First Affiliated Hospital of Chongqing Medical University, Chongqing Medical University, Chongqing, 400016 China

**Keywords:** CSF1R, Soluble CSF1R, Microgliopathy, CRL

## Abstract

Colony-stimulating factor 1 receptor (CSF1R), primarily expressed on microglia in the central nervous system (CNS), is essential for microglial homeostasis and survival. CSF1R dysfunction, due to a monoallelic mutation, causes CSF1R-related leukoencephalopathy (CRL), a primary microgliopathy. CSF1R undergoes proteolytic cleavage to release soluble CSF1R (sCSF1R), which is decreased in the serum of CRL patients. However, the biological function of sCSF1R remains unknown. Here, we found that sCSF1R alleviated cognitive impairment and anxiety-like behavior in *Csf1r*^*+/−*^ mice. Importantly, we identified CSF1R as a target binding protein of sCSF1R on microglia. Notably, sCSF1R inhibited the activation and inflammatory factor expression of *Csf1r*^*+/−*^ microglia by reducing the phosphorylation of CSF1R (Y723) and NF-κB (S468 and S536). These results demonstrate that sCSF1R exerts neuroprotective effects by binding membrane-bound CSF1R and inhibiting pathological microglial activation by inhibiting the nuclear translocation of NF-κB. These findings identify sCSF1R as a potential therapeutic agent for CRL.

**Supplementary Information:**

The online version contains supplementary material available at 10.1186/s12974-025-03648-4.

## Introduction

Microglia are resident innate immune cells in the brain and play crucial roles, including synaptic pruning, clearance of cellular debris, and neuroinflammation, in the central nervous system (CNS) [1]. Colony-stimulating factor 1 receptor (CSF1R), a member of the class III transmembrane tyrosine kinase receptor family, is primarily expressed on microglia in the adult brain and is essential for microglial proliferation, differentiation, survival, and homeostasis [2].

Previous studies have established that dysfunctional CSF1R mediates adult-onset leukoencephalopathy with axonal spheroids and pigmented glia (ALSP) [3, 4], a neurodegenerative leukodystrophy primarily characterized by dementia [5]. Diseases caused by mutations in CSF1R have recently been nominated as CSF1R-related leukoencephalopathy (CRL) [6] or CSF1R-related disorder (CSF1R-RD) [7, 8], a subtype of ALSP. Chitu et al. demonstrated that *Csf1r* heterozygosity in mice is necessary and sufficient for the development of the neurodegenerative characteristics observed in CRL patients [9], indicating that it is a primary microgliopathy. Our previous research revealed that *Csf1r* haploinsufficiency results in early impairment of synaptic plasticity and aberrant activation of microglia in *Csf1r*^+/−^ mice [5]. These results indicated that the *Csf1r*^+/−^ mouse model can simulate the clinical phenotypes observed in CRL.

Previously, we have elucidated that CSF1R is cleaved by the disintegrin and metalloproteinase 17 (ADAM17) [8] leading to the release of the N-terminal fragment, namely soluble CSF1R (sCSF1R). However, the biological functions of sCSF1R remain unclear. Importantly, our recent research showed that sCSF1R was dramatically reduced in the peripheral serum of CRL patients and mouse models [8]. Therefore, we speculated that sCSF1R might play a protective role in the pathogenesis of CRL. Further behavioral and cellular tests may be conducted to explore whether sCSF1R could serve as a therapeutic target for treating these diseases.

In this study, we found that delivery of purified recombinant human sCSF1R into mouse brains could reverse the impaired behaviors in CRL model mice. We further elucidated the mechanism by which sCSF1R suppresses microglial inflammation and activation via blockade of NF-κB nuclear translocation. Our study proposes a potential therapeutic strategy for CRL by targeting the sCSF1R pathway.

## Results

### sCSF1R protects against cognitive and anxiety-like behavior of *Csf1r*^***+/−***^ mice

We previously found that sCSF1R levels significantly decreased in the peripheral serum of CRL patients and *Csf1r*^*+/−*^ mice [8], an animal model of CRL [5, 9]. Herein, we further assessed whether sCSF1R levels also decreased in the brain. We found that both full-length CSF1R and sCSF1R were reduced in the brain tissues of *Csf1r*^*+/−*^ mice (Fig. S1A-C). Meanwhile, ELISA assay showed that sCSF1R levels in the cerebrospinal fluid (CSF) from *Csf1r*^*+/−*^ mice (Fig. S1D) were markedly decreased compared to those from wild-type (WT, *Csf1r*^+/+^) mice. These intriguing findings suggest that sCSF1R has a potential protective role in the CNS.

Our previous studies have shown that 9‑ to 11‑month‑old *Csf1r*^+/−^ mice recapitulated the clinical characteristics in CRL patients [5]. To determine the potential protective effects of sCSF1R on the behavioral deficits in CRL patients, we first purified the human sCSF1R protein. The purified protein was then quantified by standard bovine serum albumin (BSA) according to a protocol established in our recent publication (Fig. S1E) [8]. Subsequently, we treated primary microglia from WT or *Csf1r*^*+/−*^ mice with varying concentrations of sCSF1R and found that sCSF1R decreased microglial viability in a dose-dependent manner (Fig. S1F).

**Fig. 1:**
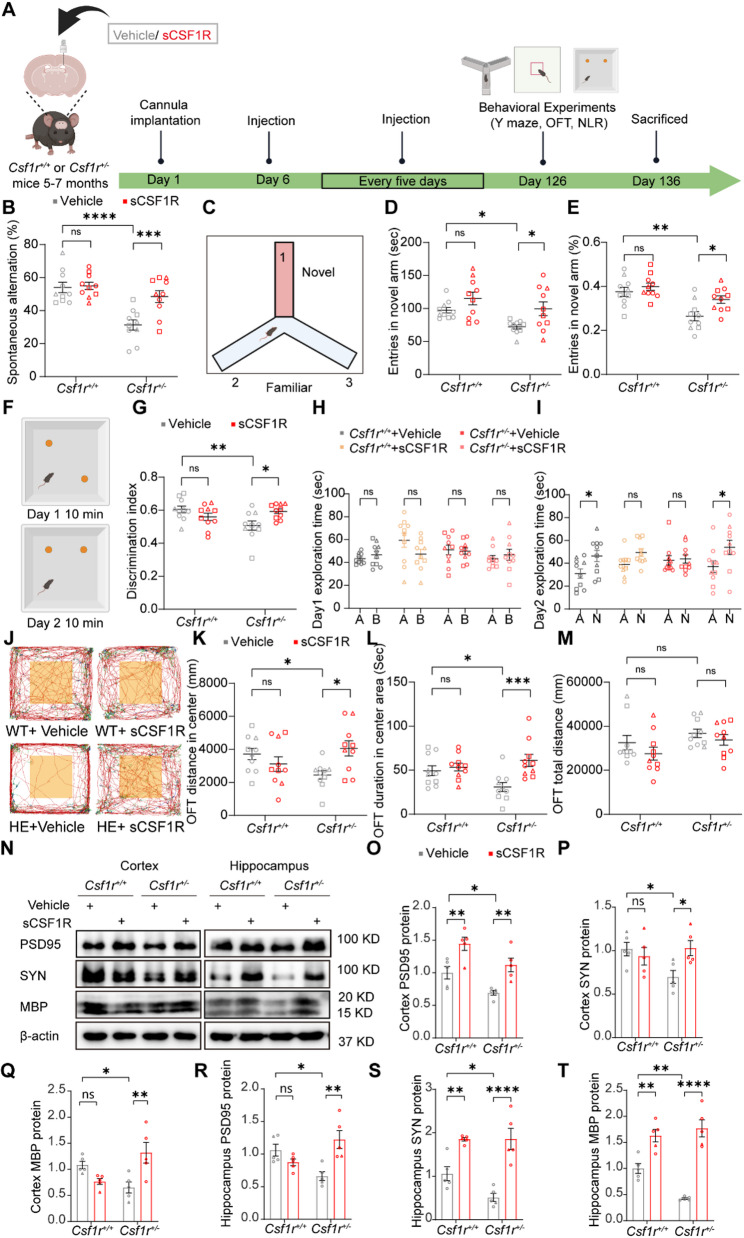
Long-term sCSF1R treatment alleviates the anxiety-like and cognitive deficits of *Csf1r*^+/−^ mice.** A** Schematic illustration of the long-term sCSF1R replenishment experimental design. Five to seven-month-old *Csf1r*^*+/+*^ or *Csf1r*^+/−^ mice were injected with Vehicle or sCSF1R protein in the lateral ventricle. **B** In the Y-maze tests, the percentages of spontaneous alternation were analyzed. **C** Schematic illustration of the novel arm recognition test. Time spent (**D**) and percentage (**E**) in the novel arm were analyzed. **F** Schematic illustration of the novel location recognition (NLR) test. In the NLR test, the discrimination index (**G**), time spent exploring objects A and B during the day 1 training phase (**H**), and time spent exploring objects A and the new location of B (object N) during the day 2 test phase (**I**) were recorded and analyzed. **J**-**M** In the open-field test, the distance (**K**) and time spent (**L**) in the center, and the total distance (**M**), were recorded and analyzed (*Csf1r*^*+/+*^ and *Csf1r*^+/−^ mice from 9 to 11-month-old, *n* = 10, two-way ANOVA). **N** Western blot detection of postsynaptic membrane protein marker (PSD95), presynaptic membrane protein marker (SYN), and myelin basic protein (MBP) in cortex and hippocampus lysates of *Csf1r*^+*/*+^ or *Csf1r*^+/−^ mice treated or not with sCSF1R. PSD95 (**O**), SYN (**P**), MBP (**Q**) protein levels in cortex quantified by densitometry, with β-actin as a loading control for comparison (*n* = 5 mice; two-way ANOVA). PSD95 (**R**), SYN (**S**), and MBP (**T**) protein levels in the hippocampus were quantified by densitometry with β-actin for comparison (*n* = 5 mice, two-way ANOVA). Data are plotted as mean ± SEM. *p < 0.05; **p < 0.01; ***p < 0.001; ****p < 0.0001; ns, not significant. Mice age, □, 9 months; ○, 10 months; △, 11 months

Given the reduced sCSF1R levels in *Csf1r*^*+/−*^ versus WT mice (Fig. S1D), we hypothesized that sCSF1R supplementation would rescue the associated behavioral deficits. To test this, we administered approximately 1 µL of sCSF1R (1 ng/µL) into the lateral ventricle of both WT and *Csf1r*^+/−^ mice every 5 days for 4 months (Fig. 1A and S2). The cognitive and anxiety-like behaviors of those mice were assessed using a series of behavioral tests. Spontaneous alternation in Y-maze tests showed that spatial working memory was impaired in *Csf1r*^+/−^ mice compared to WT mice, while sCSF1R replenishment significantly increased the percentage of spontaneous alternation in *Csf1r*^+/−^ mice (Fig. 1B). In the novel arm recognition test, *Csf1r*^+/−^ control mice (*Csf1r*^+/−^-Vehicle) spent less time and percentage in the novel arm while *Csf1r*^+/−^ mice spent more time and more entries in the novel arm upon sCSF1R treatment (Fig. 1C-E). Moreover, we used a novel location recognition (NLR) test to assess the effects of sCSF1R on hippocampal function in mice (Fig. 1F). After the training phase (Fig. 1H), compared to WT mice, *Csf1r*^+/−^ mice had a worse ability to recognize objects that had been moved, as evaluated by the discrimination index (Fig. 1G), which was alleviated in sCSF1R-treated *Csf1r*^+/−^ mice (Fig. 1I) in the testing phase. Given that *Csf1r*^+/−^ mice exhibit an anxiety-like behavior at the age of 9 months [5], we thus examined whether sCSF1R affects this emotional deficit. Consistently, open field tests showed that *Csf1r*^+/−^ mice spent significantly less time and traveled a shorter distance in the center than WT mice, indicating an anxiety-like behavior. However, sCSF1R rescued the anxiety-like behavior of *Csf1r*^+/−^ mice (Fig. 1K and L), despite no significant difference in total movement distance among the groups (Fig. 1M).

We next investigated whether sCSF1R could reverse the synaptic-associated protein changes and alleviate the demyelination in *Csf1r*^+/−^ mice. Consistent with our previous studies, we found that compared to WT mice, PSD95, SYN, and MBP were significantly down-regulated in the cortex and hippocampus of *Csf1r*^+/−^ mice [5]. Significantly, sCSF1R treatment increased the expression of PSD95, SYN, and MBP in the cortex and hippocampus of *Csf1r*^+/−^ mice (Fig. 1N-T). Meanwhile, immunofluorescence staining also indicated a significant recovery of MBP in the hippocampal CA1 (Fig. S3A and B) and corpus callosum (Fig. S3C and D) region of the sCSF1R-treated *Csf1r*^+/−^ mice. However, Sholl analysis showed no differences in microglial process morphology between the groups (Fig. S3E and F). Collectively, these results indicated that cognitive deficiency and anxiety-like behavior in *Csf1r*^+/−^ mice can be rescued by sCSF1R replenishment.

### sCSF1R binds to mouse CSF1R and inhibits the inflammation and activation of microglia in *Csf1r*^***+/−***^ mice

**Fig. 2:**
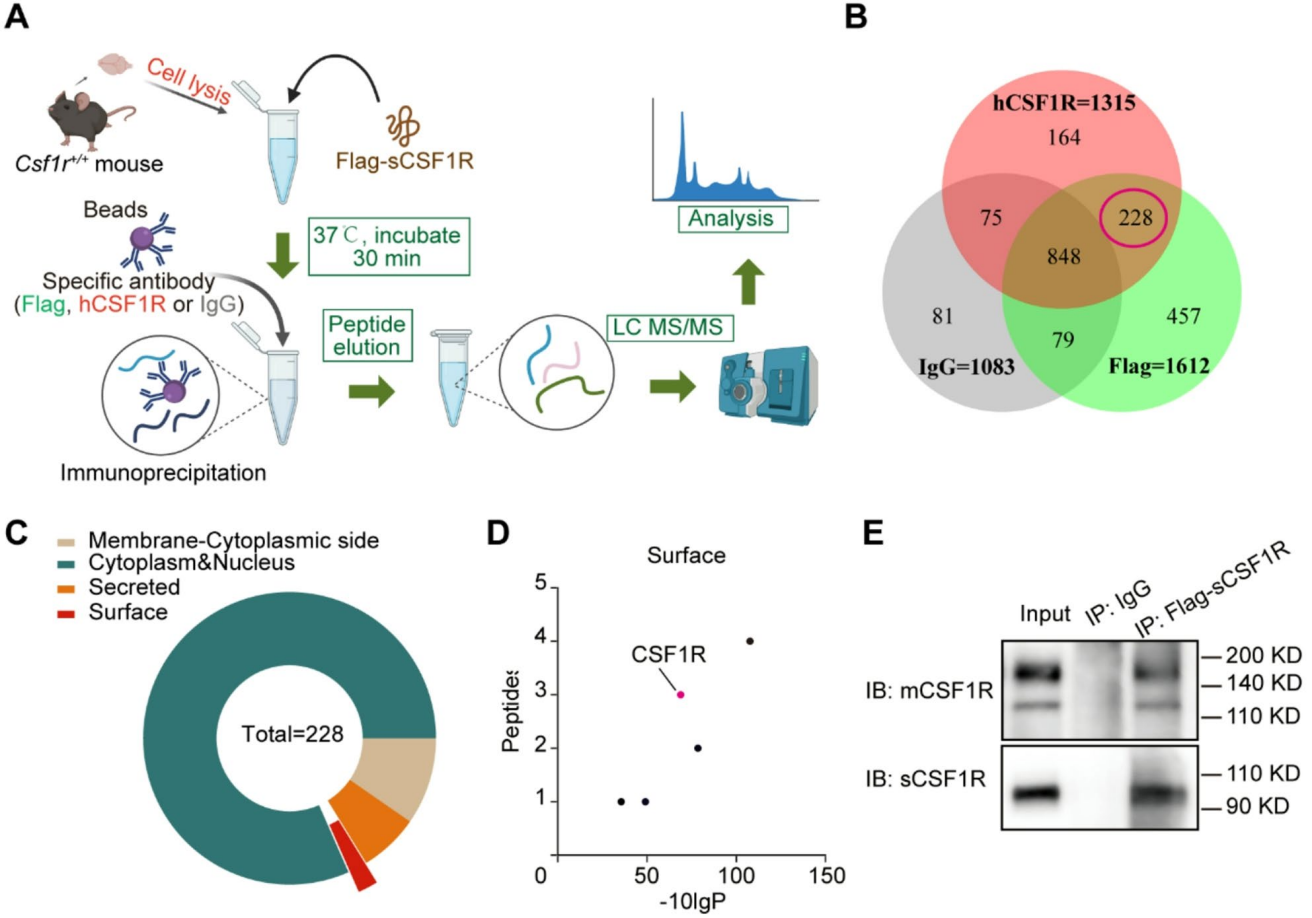
sCSF1R binds to CSF1R.** A** Schematic illustration of the Co-IP plus LC-MS/MS experimental design. **B** Venn diagram analysis revealed that 228 proteins are detected (red circle). **C** The pie chart showed the proportion of cellular subcellular localization for 228 proteins. **D** Selected candidates from LC-MS/MS assays. Details of surface proteins in 228 proteins. **E** sCSF1R protein was incubated with primary microglia cell lysate and an anti-Flag antibody for immunoprecipitation (IP), and then used to immunoblot with a mouse CSF1R antibody

Given that replenishing sCSF1R could alleviate the reduction of synaptic scaffolding proteins in *Csf1r*^+/−^ mouse brain (Fig. 1N), we next explored which membrane proteins could bind to sCSF1R in the CNS. As shown in the schematic diagram (Fig. 2A), we incubated purified sCSF1R with whole brain lysates from WT mice. Following incubation, immunoprecipitation was conducted with anti-Flag, anti-human CSF1R, or negative control IgG antibodies. A total of 1612, 1315, and 1083 proteins were quantified by LC-MS/MS in the immunoprecipitated eluates from the Flag-tag, human CSF1R, and negative control (IgG) groups, respectively (Fig. 2B, detailed information in Table. S1). Based on comparative analysis, we identified 228 candidate proteins that exhibited specific interaction with sCSF1R. Furthermore, we annotated the subcellular locations of these proteins using the UniProt database (Fig. 2C). Among these, five were specifically localized to the cell surface (Fig. 2D).

Previous studies indicated that CRL was a primary microgliopathy [10–12]. Based on the results that sCSF1R could inhibit the activation of microglia *in vivo*, CSF1R was proposed and identified to be the candidate accounting for the sCSF1R function (Fig. 2D). Using endogenous immunoprecipitation in primary microglia, we confirmed the interaction between sCSF1R and CSF1R (Fig. 2E). This finding was further supported by a cell surface labeling assay (Fig. S4).

**Fig. 3:**
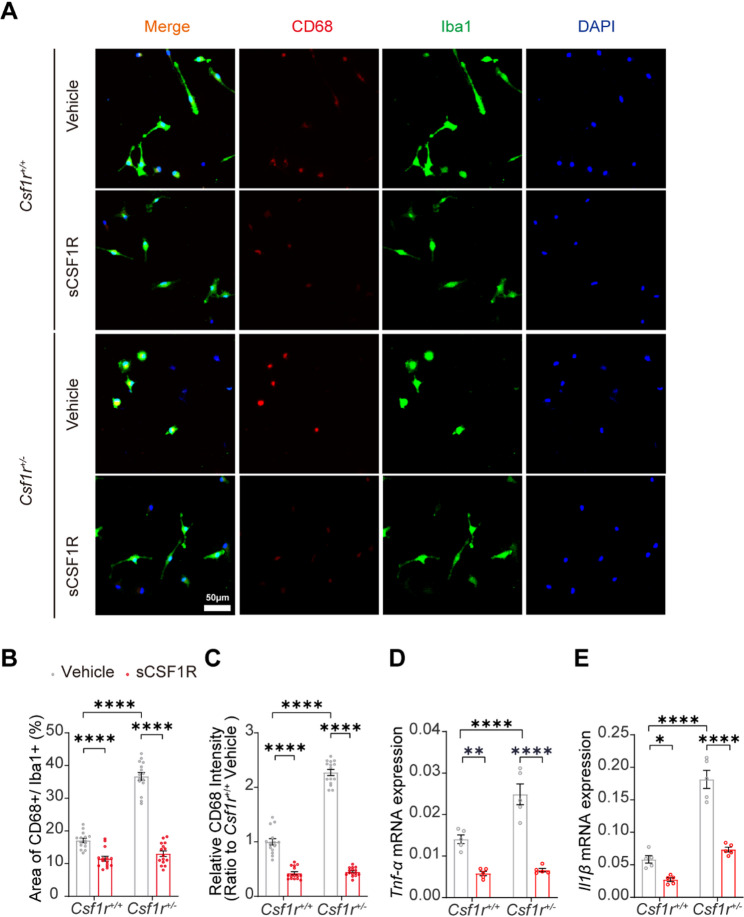
sCSF1R inhibits inflammation in primary microglia.** A** Primary microglia from *Csf1r*^*+/+*^ or *Csf1r*^+/−^ mice were treated with or without 1 ng/mL sCSF1R for 24 h, and representative images of Iba1^+^ CD68^+^ microglia are shown. Bar, 50 μm. **B-C** Statistical analysis of the percentage of CD68 (red) positive (CD68^+^) microglia relative to Iba1^+^ microglia (**B**) and the relative intensity of CD68 (**C**) per field (*n* = 15 from five independent experiments, two-way ANOVA). **D-E** *Tnf-α* (**D**) and *Il1β* (**E**) mRNA levels in primary *Csf1r*^*+/+*^ or *Csf1r*^*+/−*^ microglia treated with or without 1 ng/mL sCSF1R for 24 h were quantified by quantitative RT-PCR (*n* = 5 independent experiments). Data are presented as means ± SEM. Two-way ANOVA tests. *p < 0.05; **p < 0.01; ****p < 0.0001

**Fig. 4:**
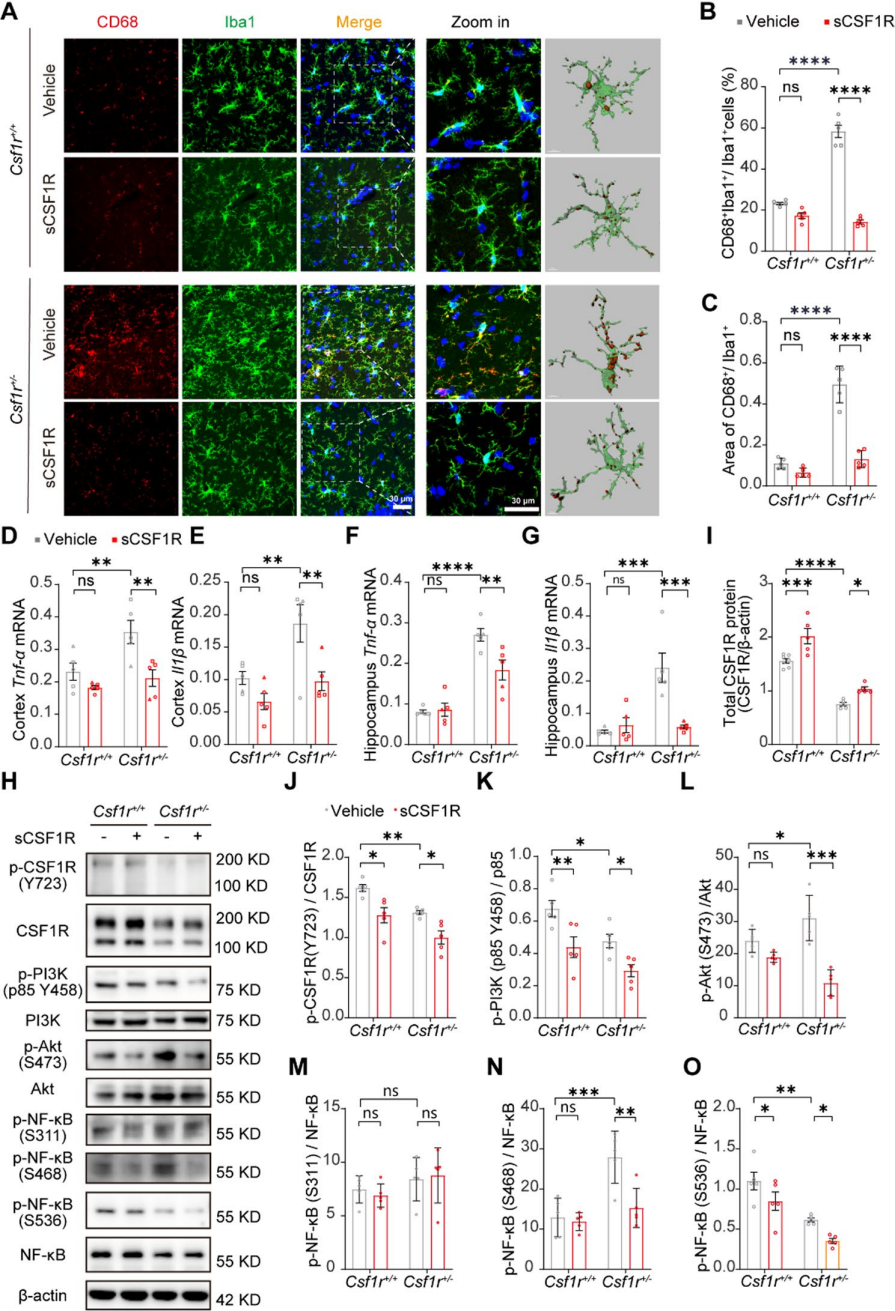
sCSF1R treatment inhibits inflammation and activation of microglia through CSF1R/PI3K/Akt/NF-κB pathway in *Csf1r*^+/−^ mice.** A** Representative images of hippocampal CA1 regions of 9 to 10-month-old *Csf1r*^*+/+*^ and *Csf1r*^+/−^ mice treated with Vehicle or recombinant sCSF1R protein. Samples were immunostained with antibodies against Iba1 (green) and CD68 (red), and stained with DAPI (blue). Statistical analysis of the percentage of CD68 and Iba1-positive (CD68^+^ Iba1^+^) microglia relative to Iba1^+^ microglia. Bar, 30 μm; zoom in bar, 5 μm. **B** and the area of CD68^+^ relative to Iba1^+^ (**C**) per field (*n* = 5 mice, two-way ANOVA). *Tnf-α* (**D**) and *Il1β* (**E**) mRNA levels in the cortex of 9 to 11-month-old *Csf1r*^*+/+*^ or *Csf1r*^*+/−*^ mice treated with Vehicle or sCSF1R were quantified by quantitative RT-PCR (*n* = 5 mice, two-way ANOVA). *Tnf-α* (**F**) and *Il1β* (**G**) mRNA levels in the hippocampus of 9 to 11-month-old *Csf1r*^*+/+*^ or *Csf1r*^*+/−*^ mice treated with Vehicle or sCSF1R were quantified by quantitative RT-PCR (*n* = 5 mice, two-way ANOVA). **H** Primary microglia from *Csf1r*^+*/*+^ or *Csf1r*^+/−^ mice were treated with Vehicle or sCSF1R for 24 h and cell lysates were analyzed by Western blotting. Representative images of Western blotting for the total and phosphorylation of CSF1R, PI3K, Akt, and NF-κB are shown. Protein levels of CSF1R (**I**) were quantified by densitometry and are presented as ratios to β-actin (*n* = 5, two-way ANOVA). Phosphorylated CSF1R at Tyr723 site (pCSF1R-Y723) (**J**) were quantified by densitometry and are presented as ratios to CSF1R (*n* = 5, two-way ANOVA). Phosphorylated PI3K (**K**) were quantified by densitometry and are presented as ratios to PI3K. Phosphorylated Akt at Ser473 site (pAkt-S473) (**L**) were quantified by densitometry and are presented as ratios to Akt (*n* = 5, two-way ANOVA). Phosphorylated NF-κB at Ser311, Ser468 and S536 site (pNF-κB-S311) (**M**), (pNF-κB-S468) (**N**), (pNF-κB-S536) (**O**) were quantified by densitometry and are presented as ratios to NF-κB (*n* = 5, two-way ANOVA). Data are plotted as mean ± SEM. *p < 0.05; **p < 0.01; ***p < 0.001; ****p < 0.0001; ns, not significant. Mice age, □, 9 months; ○, 10 months; △, 11 months

Given that *Csf1r*^+/−^ microglia were activated by producing proinflammatory factors and that CSF1R in the adult brain is crucial in maintaining microglial homeostasis [13], we then isolated primary microglia from WT and *Csf1r*^+/−^ mice to investigate the effects of sCSF1R on microglia activation and whether the protective role of sCSF1R was mediated by microglia in mouse brains. Interestingly, sCSF1R treatment significantly restored the increased intensity of CD68, a lysosome marker of microglial activation, in heterozygous CSF1R deletion (*Csf1r*^+/−^) microglia (Fig. 3A-C). Importantly, we found that, compared with WT microglia, sCSF1R treatment significantly reduced microglial hyperactivation in the hippocampal CA1 region of *Csf1r*^+/−^ mice, as assessed by CD68 immunofluorescent staining (Fig. 4A-C). However, there was no difference in Sholl analysis of microglial process among those groups (Fig. S3G).

Published works showed that elevated neuroinflammation contributes to behavioral deficits via demyelination and synaptic dysfunction [5, 14, 15]. Meanwhile, the increased mRNA levels of *Il1β* and *Tnf-α* in *Csf1r*^+/−^ microglia were significantly suppressed in response to sCSF1R treatment (Fig. 3D and E). We also observed elevated *Il1β* and *Tnf-α* transcripts in *Csf1r*^+/−^ mouse brain, which were significantly reversed by sCSF1R treatment (Fig. 4D-G).

Studies have shown that CSF1R interacts with Triggering Receptor Expressed on Myeloid Cells-2 (TREM2) [16] and that TREM2 protects against complement-mediated synaptic loss by binding to complement C1q [17]. Given the reversed expression of PSD95, SYN, and MBP in *Csf1r*^+/−^ mouse brain in response to sCSF1R, we then sought to detect the level of C1q in the mouse brain. As shown in Fig. S5A and B, C1q was elevated in the hippocampal CA1 region of *Csf1r*^+/−^ mice compared to WT controls. Corresponding to these changes, quantitative imaging showed that significantly more MBP (Fig. S5 C and D) and PSD95 (Fig. S5 E and F) puncta were found in CD68^+^ microglial structures in the CA1 region of *Csf1r*^+/−^ mouse brain compared with WT. Interestingly, sCSF1R treatment significantly reduced C1q expression and MBP/PSD95 puncta in CD68^+^ microglia in both genotypes. These results indicate that sCSF1R treatment can reduce microglial phagocytic activity in WT and *Csf1r*^+/−^ mice.

Collectively, these results suggest that sCSF1R could inhibit the inflammation and activation of microglia by binding to CSF1R in *Csf1r*^+/−^ mice.

### sCSF1R reduces the phosphorylation of CSF1R and NF-κB in *Csf1r*^***+/−***^ microglia

Next, we asked how sCSF1R inhibits microglial activation and inflammation in *Csf1r*^*+/−*^ mice. Previous studies have shown that *Tnf-α* and *Il1β* are mainly induced by NF-κB phosphorylation at Ser311, Ser468, and Ser536 [18–21], and that phosphorylation of Tyr723 on CSF1R can activate the PI3K/Akt/NF-κB pathway downstream [22–25]. We then interrogated the mechanism whereby sCSF1R binding inhibits CSF1R phosphorylation and subsequently dampens the PI3K/Akt/NF-κB signaling cascade. According to this speculation, we treated WT or *Csf1r*^*+/−*^ primary microglia with sCSF1R or Vehicle for 24 h and detected the PI3K/Akt/NF-κB pathway in those cells. In Western blot analysis, we found that, compared to WT microglia, the phosphorylation of Akt (S473) and NF-κB (S468) was up-regulated in *Csf1r*^*+/−*^ microglia (Fig. 4H, K and M). Meanwhile, phosphorylated CSF1R (Y723), PI3K, Akt (S473) and NF-κB (S468 and S536) were decreased in WT or *Csf1r*^*+/−*^ microglia in response to sCSF1R treatment (Fig. 4H, J, K, M and N) whereas the phosphorylation of STAT3, ERK or CREB was not altered (Fig. S6A-D).

Unexpectedly, total CSF1R was significantly increased in both WT and *Csf1r*^*+/−*^ microglia in response to sCSF1R, prompting the possibility that sCSF1R may stabilize the full-length CSF1R to keep the homeostasis of microglia (Fig. 4H and I).

NF-κB is a cytoplasmic transcription factor that, upon activation, undergoes nuclear translocation to drive the expression of inflammatory genes, such as *Tnf-α* and *Il1β* [26–30]. While NF-κB is a key regulator, the transcription of *Tnf-α* and *Il1β* also involves other factors including STAT3, ERK, and CREB [31–34]. We found that compared to WT microglia, the nucleus location of NF-κB (Fig. S6E and G), but not STAT3, ERK, or CREB (Fig. S6E, F, H, and I), was increased in *Csf1r*^*+/−*^ microglia. We next asked whether sCSF1R inhibited NF-κB nuclear translocation in *Csf1r*^*+/−*^ microglia. Microglia treated with sCSF1R showed a significant decrease in the location of NF-κB in the *Csf1r*^*+/−*^ microglial nucleus, suggesting that sCSF1R inhibits NF-κB entry into the nucleus (Fig. S6J and K). Correspondingly, sCSF1R also reduced the microglial NF-κB nuclear translocation *in vivo* as evidenced by NF-κB and DAPI co-staining in microglia (Fig. S6L and M).

Building on the published literatures that S468 phosphorylation suppresses S536 phosphorylation to attenuate NF-κB transcriptional activity [35, 36], we hypothesized that differential CSF1R signaling might regulate this switch. To test this, we analyzed nuclear NF-κB phosphorylation in *Csf1r*^*+/+*^ and *Csf1r*^*+/−*^ microglia. We found that *Csf1r*^*+/−*^ microglia exhibited elevated Ser536 phosphorylation in the nucleus, consistent with a mechanism of enhanced transcriptional activation. This increase was reversed by sCSF1R treatment, suggesting that CSF1R signaling, directly or indirectly, sustains the S468-phosphorylated, transcriptionally repressed state of NF-κB (Fig. S7A-D). Collectively, these observations point to a paradoxical coexistence of pro-inflammatory (elevated pNF-κB S536) and putative anti-inflammatory (*via* the pNF-κB S468 repressive pathway) signaling within CRL microglia. This bistable regulatory regime demands deeper mechanistic inquiry.

These results indicate that sCSF1R reduces CSF1R phosphorylation and NF-κB nuclear localization *in vitro *and *in vivo*.

### sCSF1R reduces the enhanced mitochondrial dynamics in *Csf1r*^***+/−***^ microglia via inhibiting the translocation of NF-κB

**Fig. 5:**
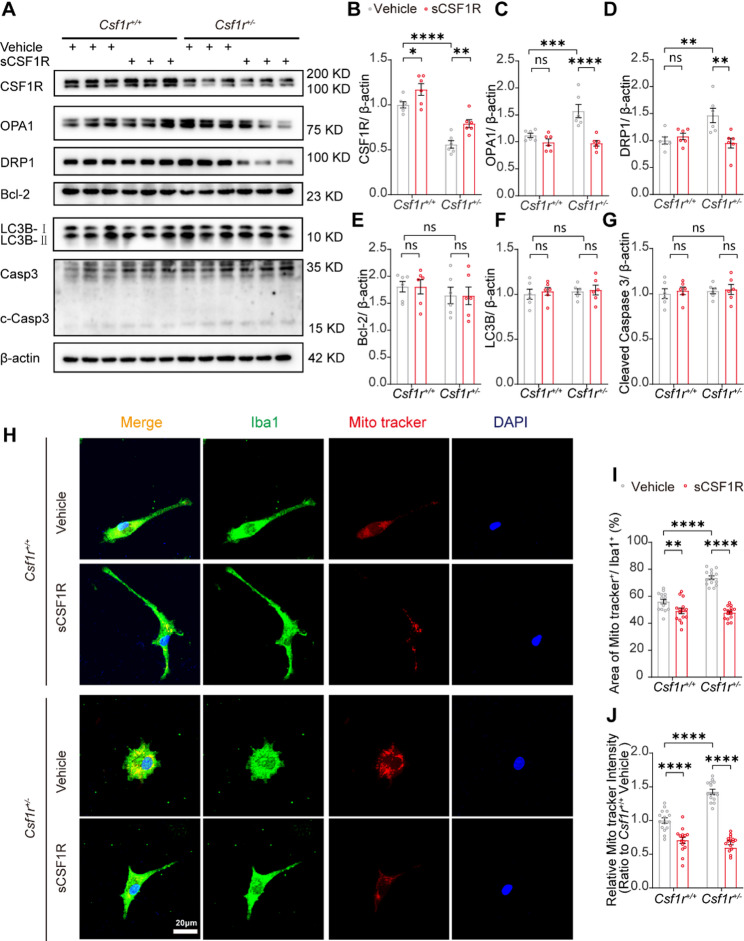
sCSF1R inhibits the mitochondrial-associated proteins in *Csf1r*^+/−^ microglia.** A** Primary microglia from *Csf1r*^+*/*+^ or *Csf1r*^+/−^ mice were treated with Vehicle or sCSF1R for 24 h and cell lysates were analyzed by Western blotting. Representative images of Western blotting for the total and phosphorylation of CSF1R, DRP1, OPA1, Bcl-2, LC3B, Caspase-3 and cleaved Caspase-3 are shown. Protein levels of CSF1R (**B**), OPA1 (**C**), DRP1 (**D**), Bcl-2 (**E**), LC3B (**F**) and cleaved Caspase-3 (**G**) were quantified by densitometry and are presented as ratios to β-actin (*n* = 6, two-way ANOVA). **H** *Csf1r*^*+/+*^ or *Csf1r*^+/−^ microglia were treated or not with sCSF1R for 24 h, and representative images of Iba1^+^ Mito-tracker^+^ microglia are shown. Statistical analysis of the area of Mito-tracker (red) positive (Mito-tracker ^+^) microglia relative to Iba1^+^ microglia (**I**) and the relative intensity of Mito-tracker (**J**) per field (*n* = 15 from five independent experiments, two-way ANOVA). Bar, 20 μm. Data are plotted as mean ± SEM. *p < 0.05; **p < 0.01; ***p < 0.001; ****p < 0.0001; ns, not significant

Next, we sought to investigate the mechanisms by which sCSF1R inhibits microglial viability and activation (Fig. S1F and Fig. 4). We supposed that microglia may undergo apoptosis, autophagy, or decreased metabolism. Bcl-2 and Caspase-3 are the marker of apoptosis [37, 38], and LC3B is the marker of autophagy [39]. Proteins associated with mitochondrial activity, such as dynamin-related protein 1 (DRP1) and optic atrophy protein 1 (OPA1) [40–43], were also the target genes regulated by NF-κB [44–48]. Thus, we performed Western blot analysis to determine whether these key regulators of mitochondrial fusion and fission in WT or *Csf1r*^*+/−*^ primary microglia were affected by sCSF1R. Treatment with sCSF1R significantly reversed the increase of OPA1 and DRP1 expression in *Csf1r*^*+/−*^ microglia (Fig. 5A, C, and D), whereas Bcl-2, LC3B, and cleaved caspase-3 were not affected by sCSF1R in *Csf1r*^*+/−*^ microglia (Fig. 5A, E, F and G). Additionally, we used Mito-Tracker 594 to observe the mitochondrial dynamics in WT and *Csf1r*^*+/−*^ primary microglia. We found that the area and intensity of mitochondrion in *Csf1r*^*+/−*^ microglia were significantly increased compared to WT cells, whereas sCSF1R treatment led to a reduction of mitochondrial dynamics in WT and *Csf1r*^*+/−*^ microglia (Fig. 5H-J). Moreover, sCSF1R treatment could reduce the expression of microglial mitochondrial-related proteins DRP1 and OPA1, which were increased in the CA1 region of *Csf1r*^+/−^ mouse brain (Fig. S8A-C). Subsequently, we used JSH23, an inhibitor of NF-κB nuclear translocation [49], to treat microglia for 24 h. As shown in Fig. S8D-I, JSH23 reduced DRP1 and OPA1 in *Csf1r*^*+/−*^ microglia and increased the CSF1R in both WT and *Csf1r*^*+/−*^ microglia. Meanwhile, this change was not additive to the sCSF1R. These results revealed that sCSF1R reduces the enhanced mitochondrial dynamics in *Csf1r*^+/−^ microglia by inhibiting NF-κB translocation, suggesting that this is the molecular mechanisms underlying sCSF1R-mediated inhibition of microglial viability and activation.

### sCSF1R plays an antagonistic role in the classical IL34/CSF1R pathway

In our Co-IP-LC-MS/MS data, Interleukin-34 (IL-34), a known CSF1R ligand, was also a candidate target protein of sCSF1R (Fig. 2C and Supplementary Table 1). Literatures have shown that IL34-CSF1R axis plays an essential role in microglial survival, CNS development, and neurodegeneration [50, 51]. Does sCSF1R compete with IL34 in binding to CSF1R? We then carried out Co-IP in FBS free medium and found that sCSF1R bound to IL34 and the N-terminal fragment of mouse CSF1R (NTF-HA, 1-505aa) (Fig. S9A). Moreover, we used the same concentration of mIL34 (10 pM, approximately 0.34 ng/mL) and sCSF1R (10 pM, 1 ng/mL) to treat microglia from WT and *Csf1r*^*+/−*^ mice for 24 h. As shown in Fig. S9B-H, mIL34 activated the CSF1R signaling pathway, and this activation was abolished by sCSF1R. These results indicate that sCSF1R binds to mIL34 and mCSF1R to form a trimer complex and that sCSF1R acts as a functional antagonist in the classical IL34/CSF1R pathway.

## Discussion

CSF1R, a transmembrane receptor tyrosine kinase, is necessary for the development, survival, and homeostasis of microglia in the brain. Our previous study demonstrated a stepwise approach of human CSF1R cleavage releasing sCSF1R. The full-length form of CSF1R is expressed on microglia, and sCSF1R is shed by ADAM17-mediated cleavage [8, 52]. However, its physiological and pathological functions, including its relationship with CRL, remain unclear in humans. This study highlights for the first time the physiological role of sCSF1R in the brain. By purifying sCSF1R from the HEK293T cell line and treating primary microglia with sCSF1R, we found that sCSF1R can inhibit microglial activation and proinflammation in *Csf1r*^*+/−*^ mice, suggesting that sCSF1R may be therapeutic for diseases involving microglial dyshomeostasis. CRL is a neurodegenerative white matter disease caused by CSF1R mutations, characterized by excessive microglial activation [53–56]. Our study demonstrated that replenishing sCSF1R in *Csf1r* haploinsufficient mouse model of CRL can reverse reductions in synaptic scaffolding proteins and cognitive impairments in *Csf1r*^*+/−*^ mice. Mechanistically, using an LC-MS/MS assay, we identified a candidate surface protein, CSF1R, which is mainly expressed on microglia in the brain [13]. It is important to note that the mouse cohort ranged in age from 9 to 11 months at the time of analysis. We acknowledge that the sample size was insufficient to support a well-powered, stratified analysis or to include age as a covariate. As a result, we cannot rule out the possibility that age differences may have influenced the observed outcomes. Future studies utilizing animals within a narrower age range will help to clarify these findings and strengthen the conclusions.

Note that in primary microglia from *Csf1r*^+/−^ mice, CSF1R protein levels are significantly reduced due to haplo-deficiency, however, the downstream NF-κB signaling pathways of CSF1R are activated explicitly in microglia from *Csf1r*^+/−^ mice. We speculate that reduced CSF1R expression might trigger compensatory upregulation of alternative receptors (e.g., TREM2) or adaptor proteins (e.g., PI3K subunits) that converge on NF-κB pathways. We confirmed TREM2 upregulation in *Csf1r*^+/−^ microglia via qPCR (Fig. S9I-J), suggesting a compensatory mechanism. This finding aligns with our previous work, which demonstrated increased *Trem2* mRNA in primary microglia following *Csf1r* knockdown [16] and revealed that TREM2 and CSF1R can form complexes and mutually regulate their expression. Furthermore, we have also reported that TREM2 stabilizes β-catenin by inhibiting its degradation via the Akt/GSK3β pathway [57], outlining a key downstream signaling axis. The intricate relationship between these pathways presents a compelling question for future investigation. The *Csf1r*^+/−^ genotype may polarize microglia toward a pro-inflammatory state (e.g., via TLR crosstalk), bypassing CSF1R’s canonical regulation. This paradoxical activation could reflect nonlinear signaling thresholds or cell-state-specific rewiring, highlighting the complexity of CSF1R biology in microglia. Thus, whether downstream signaling be further enhanced upon complete knockout of CSF1R requires further experimental verification.

Previous research revealed that the phosphorylation levels of S311 and S468 sites of NF-κB protein in the hippocampal CA1 region of a status epilepticus rat model were significantly increased through the PI3K/Akt/NF-κB pathway [18, 19]. Meanwhile, phosphorylation of CSF1R on Try723 can bind the p85 subunit of PI3 kinase [58]. In the current study, the effects of sCSF1R treatment on the PI3K and NF-κB pathways may occur independently. Interestingly, sCSF1R can reduce the dynamics of mitochondrial by inhibiting the phosphorylation of CSF1R on Try723 and the phosphorylation of NF-κB on Ser468 and Ser536 in microglia, whereas sCSF1R can increase the protein level of CSF1R. The observed concurrent elevation of S468 and S536 phosphorylation of NF-κB protein in *Csf1r*^*+/−*^ microglia presents an apparent paradox. However, the increased level of phosphorylated NF-κB in the nucleus unequivocally confirms its enhanced transcriptional activity in *Csf1r*^*+/−*^ microglia. The concurrent increase in total S468 and S536 levels may suggest that *Csf1r*^*+/−*^ microglia exist in a dynamic equilibrium between pro-inflammatory and anti-inflammatory states. In this scenario, continuously activated NF-κB translocates into the nucleus to initiate transcription, while negative feedback mechanisms simultaneously promote its nuclear export when transcription is suppressed, leading to its accumulation in the cytoplasm. The marked contrast between the whole-cell and nuclear levels of phosphorylated NF-κB implies that these microglia are subject to highly dynamic and compartment-specific regulation. This compelling phenomenon undoubtedly merits further in-depth investigation in future studies. Meanwhile, mitochondrial staining showed that the mitochondria of *Csf1r*^*+/−*^ microglia were highly active, with significantly elevated levels of mitochondrial fission and fusion proteins. However, sCSF1R treatment reversed the enhanced mitochondrial dynamics of *Csf1r*^*+/−*^ microglia. While enhanced mitochondrial dynamics can be cytoprotective under acute stress, the sustained upregulation of DRP1/OPA1 and increased mitochondrial intensity in *Csf1r*^*+/−*^ microglia align with pathological hyperactivation observed in neurodegenerative disorders [59, 60]. This phenotype may reflect failed adaptation to chronic metabolic demands, ultimately exacerbating neuroinflammation and synaptic damage. Additionally, DRP1 and OPA1 were also the target genes regulated by NF-κB [44–48]. Therefore, we treated microglia with JSH23, a specific NF-κB inhibitor, and obtained results identical to those with sCSF1R treatment. Meanwhile, we found that JSH23 increased CSF1R levels in both WT and *Csf1r*^*+/−*^ microglia. Based on our data, we speculate that JSH23 does not directly modulate *Csf1r* mRNA levels but rather indirectly influences them through downstream events triggered by its inhibition—such as altered production of inflammatory mediators. However, whether the *Csf1r* mRNA level was directly affected by JSH23 in primary microglia or indirectly by the subsequent events following JHS23 inhibition remains to be further investigated.

It is noteworthy that previous studies reported detecting sCSF1R in the culture medium of primary macrophages from teleost fish [61] and goldfish [62], and they found that sCSF1R might play an inhibitory role in myeloid cell inflammation or proliferation. Recently, one study also identified a soluble form of CSF1R protein (sCSF1R) by plasma proteome profiling. The authors found that compared to the 10 healthy controls, plasma sCSF1R was significantly higher in 104 patients with Langerhans cell histiocytosis (LCH), a rare hematologic neoplasm characterized by the proliferation of Langerhans-like cells, indicating the clinical importance of the plasma sCSF1R at diagnosis and during follow-up in pediatric LCH patients [63].

Canonical CSF1R activation occurs through binding to its cognate ligands: CSF1 or IL34. Given that our LC-MS/MS analysis specifically identified IL-34 (but not CSF1) as an interacting partner, we further demonstrate that sCSF1R forms a tripartite complex with both IL-34 and the N-terminal fragment of mouse CSF1R (mNTF-HA, 1–505 aa). Importantly, functional characterization revealed that sCSF1R acts as a potent antagonist of the IL-34/CSF1R signaling pathway. While our Co-IP-LC-MS/MS analysis did not detect direct binding between sCSF1R and CSF1, we cannot conclusively exclude this potential interaction. This possibility warrants further investigation through additional experimental approaches. Studies have demonstrated that microglial dyshomeostasis was the primary cause of CRL. Correspondingly, PLX5622, a CSF1R inhibitor, was used to treat adult *Csf1r*^*+/−*^ mice, preventing microglial dyshomeostasis and behavioral deficits [64]. Our published study showed that minocycline, a microglial inhibitor, can ameliorate behavioral impairment and CRL pathology in CSF1R-haplo‑insufficient male mice [5]. Consistent with these published studies, the present study supports the feasibility of sCSF1R as an inhibitor to maintain microglial homeostasis.

In this study, other proteins were identified by LC-MS/MS, including the broad-spectrum neuron-specific protein Thy1 and the L1CAM protein. It’s interesting to investigate whether sCSF1R interacts with Thy1 and L1CAM and to explore the biological function of this interaction in future studies. L1CAM is explicitly expressed at the axon initial segment (AIS) of GABAergic neurons and plays a vital role in the initiation of action potentials [65]. Meanwhile, studies have shown that L1CAM is also involved in the extension of neuronal axons during early development and in maintaining neuronal function in adulthood [66, 67]. Therefore, we speculate that sCSF1R may also play a crucial role in neurons by binding to L1CAM, a hypothesis that warrants further research.

**Fig. 6:**
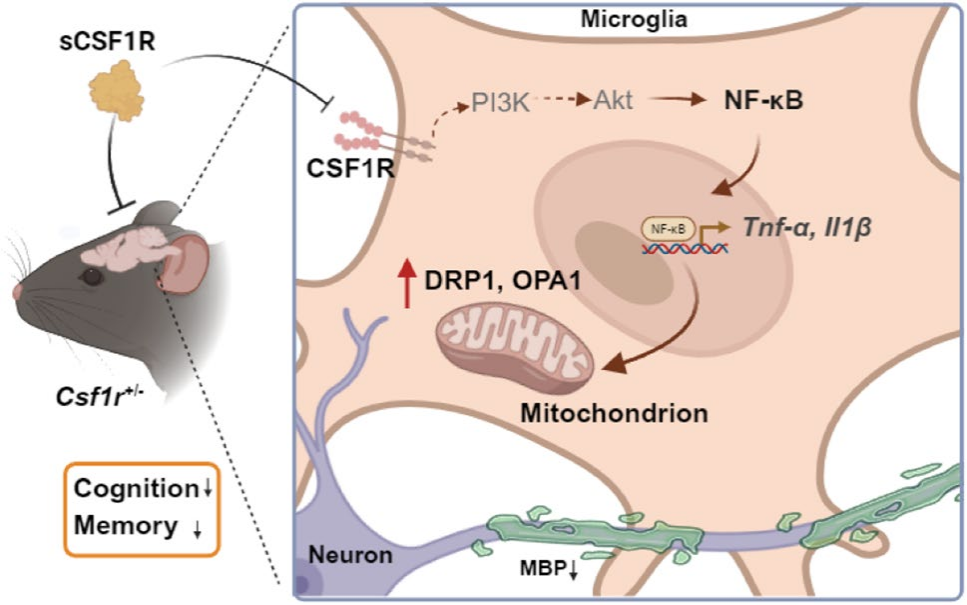
Mechanistic schematic of sCSF1R-mediated amelioration of CRL pathology in *Csf1r*^+/−^ mice. In CRL pathology, CSF1R haploinsufficiency causes NF-κB hyperactivation, driving microglial dysfunction. Soluble CSF1R exerts its therapeutic effects by directly binding to CSF1R and inhibiting downstream NF-κB nucleus translocation

In summary, this study demonstrates that the NF-κB pathway is hyperactivated in *Csf1r*^+/−^ microglia, driving the production of proinflammatory cytokine (e.g., IL-1β, TNFα) and contributing to behavioral deficits in the CRL model. Critically, our findings demonstrate that sCSF1R treatment effectively restores microglial homeostasis by binding to CSF1R, leading to partial but significant amelioration of cognitive and motor impairments in the CRL mouse model (Fig. 6). To our knowledge, this is the first demonstration that sCSF1R can rescue both microglial dysfunction and behavioral deficits in a CSF1R-deficient model. These findings provide preclinical evidence for the therapeutic potential of sCSF1R in CSF1R-related neurodegenerative disorders, though further studies are needed to characterize its long-term effects thoroughly.

## Materials and methods

### Plasmids

Human full length CSF1R (NM_001349736.2) was cloned into pIRES2-EGFP vectors, and a GGGS linker, 6×His tag and a Flag tag were inserted between 19 and 20 aa (after signal peptide). Mouse full length CSF1R (NM_001037859.2) was cloned into pIRES2-EGFP vectors, a GGGS linker and a HA-tag were inserted before the stop codon. Mouse IL34 (NM_001135100.2) was cloned into pIRES2-EGFP vectors, a GGGS linker and a MYC-tag were inserted before the stop codon. N-terminal fragment of mouse CSF1R (NTF) plasmid was constructed with KOD -Plus- Mutagenesis Kit (Cat. SMK-101, TOYOBO).

### Reagents and antibodies

Nickel-charged resin (Ni-NTA Superflow) was purchased from Qiagen (30430, Germany). Anti-mouse CD68 (RRID: AB_322219) was from Bio-Rad. Anti-Iba1 (RRID: AB_2820254), anti-PSD95 (RRID: AB_2292883), Anti- Synapsin-1 (RRID: AB_2616578), anti-human CSF1R (RRID: AB_2799725), anti-NF-κB (RRID: AB_10859369), anti-Phospho-NF-κB (Ser536) (RRID: AB_331284), anti-Phospho-CSF-1R (Tyr723) (RRID: AB_2085229), anti-Phospho-PI3K (RRID: AB_2895293), anti-PI3K (RRID: AB_2165248), anti-Phospho-STAT3 (RRID: AB_2491009), anti-Phospho-CREB (RRID: AB_2561044), anti-Phospho-ERK (RRID: AB_2315112), anti-DRP1 (RRID: AB_10950498), anti-Bcl-2 (RRID: AB_1903907), anti-LC3B (RRID: AB_915950), Rabbit IgG control for immunoprecipitation (RRID: AB_1031062), were all bought from Cell Signaling Technology. Anti-mouse CSF1R (RRID: AB_2927559), anti-Myelin Basic Protein (RRID: AB_297797), Anti-mouse C1q antibody (RRID: AB_2732849), goat anti-rabbit IgG, (H + L) (RRID: AB_10371940), goat anti-mouse IgG (H + L) (RRID: AB_2755049) and Alexa Fluor 647 donkey anti-rat IgG H&L (RRID: AB_150155), Alexa Fluor 488 goat anti-Rabbit IgG H&L (RRID: AB_2630356) were all from Abcam. Anti-OPA1 (RRID: AB_399889) was purchased from BD Biosciences. Anti-GFAP antibody (RRID: AB_2109646), Anti-Flag tag antibody (RRID: AB_11232216) and Anti-HA tag antibody (RRID: AB_11042321) were bought from Proteintech Group. Anti-MYC tag antibody (RRID: AB_2770408) was bought from ABclonal. Anti-Phospho-NF-κB (Ser311) (Cat: AF5878) was bought from Beyotime. Anti-Phospho-NF-κB (Ser468) (Cat: K011428P) was bought from Solarbio. DAPI (D9542) and poly-L-lysine (PLL, P6282) were purchased from Sigma Aldrich. TRIzol (CW05800s) and 100 × phosphatase inhibitor (CW2383S) were purchased from CWBIO. ReverTra Ace qPCR RT Master Mix (R323-01) and HamQ Universal SYBR qPCR Master Mix (q711-03) were all from Vazyme. DMEM (11965126) and 1% penicillin/streptomycin (15140122) were all bought from Gibco. Fetal bovine serum (FBS, HK026) was from NTC. RIPA buffer (AR009550) and BCA Protein Assay Kit (AR0197) were from BOSTER. 50 × protease inhibitor (00329505) was purchased from Roche. Super ECL Detection Reagents (36208ES76) were from Yeasen. Granulocyte–macrophage colony-stimulating factor (GM-CSF) was from R&D Systems. MitoTracker™ Red CMXRos was purchased from Invitrogen(M7512). NE-PER Nuclear and Cytoplasmic Extraction Reagents were purchased from Thermo Fisher (78833). Poly-L-lysine hydrobromide (PLL, MCE, HY-126437 H) and JSH23 were purchased from MedChemExpress (MCE, HY-13982).

### Cell culture, transfection, protein expression, purification, and silver staining

HEK293T cells were cultured in Dulbecco’s modified Eagle’s medium (DMEM) with 10% fetal bovine serum (FBS) and 1% P/S. Cells were cultured at 37 °C in a 5% CO2 atmosphere. HEK293T cells was seeded in 150 mm plate and grown to 8 × 10^6^ cells, 50 µg the human CSF1R-pIRES2-EGFP plasmid or empty vector plasmid was transiently transfected into HEK293T cells with Polyethylenimine Linear MW25000 (PEI MW25000, Yeasen, 40815ES03) as a ratio of 1:4. After 18 h, HEK293T was washed by 1×PBS for three times and cell culture medium was replaced with DMEM (without FBS) cultured for another 48 h and harvested. Proteins were purified by Nickel-charged resin. Purified proteins were quantified by silver staining. Purified sCSF1R was filtered by a 0.22 μm polyethersulfone syringe filter and dialyzed several times (SLGPR33RS, Millipore) for subsequent experiments. Bohlen CJ et al. reported that fetal bovine serum can have significant effects on primary microglia [68]; accordingly, we used a serum-free condition in our experiments.

## Enzyme-linked immunosorbent assay

The concentrations of sCSF1R in CSF samples were analyzed by Enzyme-Linked Immunosorbent Assay (ELISA, Cat. EK0808, Boster) according to the manufacturer’s recommendation.

### Cell viability

Cell viability was evaluated by Cell Counting Kit-8 (CCK8) assay. A density of 4 × 10^4^ primary microglia per well were seeded into 96-well culture plates and incubated overnight. Cells were treated with different concentrations of sCSF1R (serum free DMEM) for 24 h, and the viability was measured using CCK8 at 450 nm.

### Mice

All animal experiments were approved by the Animal Ethics Committee of the Xiamen University and were conducted in compliance with all relevant ethical regulations for animal testing and research. A CSF1R heterozygous (*Csf1r*+/−; C57BL/6) mouse was generated using the CRISPR/Cas9 gene-editing system as described previously [5]. CSF1R homozygous (HO, *Csf1r*^−/−^) mouse, CSF1R heterozygous (HE, *Csf1r*^+/−^) mouse and CSF1R wild-type (WT, *Csf1r*^+/+^) mouse were acquired by self-matching of *Csf1r*^+/−^ mouse. WT or HE male mice at 5 to 7 months of age were implanted with a cannula. Six days later, they were injected with control (Vehicle purification residual) or sCSF1R protein into the right lateral ventricle every 5 days (10 mice per group). 120 days after injection, mice were subjected to behavior tests. Mice were then anesthetized and perfused with ice-cold PBS. Samples were harvested for biochemical or histological analysis. After behavioral test, mice were sacrificed for IHC (*n* = 5), Western blot and RT-PCR (*n* = 5). We did perform a power analysis to determine the appropriate sample sizes.

### Primary microglia culture and treatment

Primary microglia were isolated and cultured as described previously [5]. Cells were cultured at a density of 1.5 × 10^4^ cells/well, 1.5 × 10^6^ cells/well, or 7 × 10^5^ cells/well in 96-well, 6-well, or 12-well plates and treated with or without sCSF1R (1 ng/mL) for 24 h (without FBS) for subsequent experiments.

### RNA isolation and real-time quantitative PCR analysis

Total RNA was extracted using TRIzol reagent and 1 µg RNA was reverse-transcribed into complementary DNA (cDNA) using HiScript III All-in-one RT SuperMix (R333, Vazyme). Target genes were amplified using ChamQ Universal SYBR qPCR Master Mix (Q711, Vazyme) on the LightCycler 480 (Roche, Mannheim, Germany). The mRNA level of the target gene was calculated by the relative amount of the target gene ratio to that of the internal control *Actb* (2 − ΔΔCT). The primer sequences were as follows: *Tnf-α*- Forward: GTCTACTGAACTTCGGGGTGAT, *Tnf-α*-Reverse: CTGAGTGTGAGGGTCTGGGC; *Il1β*: Forward: CAGGCAGGCAGTATCACTCATTG, *Il1β*-Reverse: GCTTTTTTGTTGTTCATCTCGGA; *Trem2*: Forward: TGCTGGCAAAGGAAAGGTG, *Trem2*-Reverse: GTTGAGGGCTTGGGACAGG; *Csf1r*: Forward: GGTTGTAGAGCCGGGTGAAA, *Csf1r* -Reverse: AAGAGTGGGCCGGATCTTTG; *Actb*-Forward: AGCCATGTACGTAGCCATCCA, *Actb*-Reverse: TCTCCGGAGTCCATCACAATG.

### Total mouse brain immunoprecipitation and LC-MS/MS

The total brain of a five-month-old WT male mouse was dissociated by 1% TNEN (150 mM NaCl, 50 mM Tris-HCl pH 8.0, 2 mM EDTA, 1% NP-40) lysis buffer at 4℃ for 1 h after a mechanical dissociation. The lysate was centrifuged at 12,000 rpm, 4℃ for 15 min, and the supernatant was collected. 6 µg of sCSF1R was mixed with the supernatant and incubated for 1 h at 37℃. The mixture was divided into three equal parts and added to the incubated magnetic bead antibody mixture (IgG control, human CSF1R, Flag-tag) for immunoprecipitation. After elution, the sample was washed 5 times with 1×PBS to remove the detergent, and then eluted with 8 M Urea. Samples were detected and analyzed on timsTOF Pro (Bruker) equipped system.

### Immunoprecipitation of primary microglial cells

Primary microglia were harvested as a previous study [5]. A total of 8 × 10^6^ cells were dissociated by 1% TNEN lysis buffer at 4℃ for 1 h after a mechanical dissociation. Lysate was centrifuged at 12,000 rpm, 4℃ for 15 min and the supernatant was collected and divided into three equal parts for Input, IgG control, or Flag-tag. Purified sCSF1R (2 µg) was mixed with the supernatants, which were incubated with magnetic bead antibody mixture (IgG control or Flag-tag) overnight for immunoprecipitation. After the elution step, samples were analyzed by Western blot.

### Immunoprecipitation of serum-free conditioned medium from HEK293T cells

Plasmids of Vehicle (pIRES2-EGFP), sCSF1R-Flag, mIL34-MYC, or N-terminal fragment of mouse CSF1R (NTF-HA, 1-505aa) were transfected into HEK293T cells using Polyethylenimine Linear MW25000. After 18 h, cells were washed 3 times with 1×PBS to remove residual serum. Medium was replaced with serum-free DMEM for an additional 48 h to collect secreted proteins. Serum-free conditioned medium was collected and concentrated using a 10 kDa ultrafiltration. The concentrated medium was divided into three equal parts: Input (direct analysis), IgG control (non-specific antibody), and Flag-tag IP (anti-Flag magnetic beads) for immunoprecipitation. After elution, samples were detected by Western Blotting assay using IP-grade antibodies (Anti-Flag, Anti-MYC, or Anti-HA).

### Cell surface labeling assay in HEK293T cells

The cell surface labeling assay was conducted according to an established protocol [69], with specific modifications. HEK293T cells were transfected with a control (vehicle) plasmid or a mouse CSF1R plasmid, each co-expressing an mCherry fluorescent tag. Transfected cells were then trypsinized and reseeded onto poly-L-lysine hydrobromide (PLL)-coated glass slides. After adherence, cells were washed extensively with cold PBS and incubated in serum-free DMEM supplemented with sCSF1R for 15 min at 4 °C to permit ligand binding. Cells were subsequently fixed with 4% paraformaldehyde (PFA) for 20 min at 4 °C. For surface labeling of CSF1R, fixed cells were incubated with an anti-Flag primary antibody (prepared in 5% BSA without detergent) for 30 min at 4 °C, followed by incubation with a corresponding secondary antibody for 20 min at room temperature. After a final series of PBS washes, coverslips were mounted and prepared for fluorescence microscopy and quantitative analysis.

### Western blotting

Samples were homogenized and incubated in RIPA Lysis and Extraction Buffer (YEASEN), supplemented with Protease and Phosphatase Inhibitor Cocktail (HY-K0010, HY-K0021, MedChemExpress). Protein concentrations were determined using the BCA Protein Assay Kit (BOSTER) according to the manufacturer’s instructions. Equal amounts of total proteins were resolved by SDS-PAGE electrophoresis and transferred to PVDF membranes (Millipore, IPVH00010). After blocking, the membranes were blotted using a primary antibody (usually 1:1,000) and detected with HRP-conjugated secondary antibody (1:10,000). Proteins were visualized using ECL Western blotting detection reagents (Yeasen). Immunoreactive bands were quantified using ImageJ software.

### Behavior tests

Before behavioral experiments, all the mice were gently touched for 3 days to avoid stress responses and placed in the test chamber for more than 30 min to adapt to the environment. All the behavior tests were performed and analyzed using CleverSys TopScanLite animal behavior analysis system (USA).

### Y‑maze and modified Y-maze test

For the Y-maze test, as previously reported [70, 71], each test mouse was placed in the center of a “Y” shaped chamber [30 cm (L) × 6 cm (W) × 15 cm (H)] and allowed to enter each arm freely for 5 min. The sequence of arm entries and the total numbers of arms entered by each mouse were recorded. The percentage of alternation was calculated as the ratio of consecutive specific arm entries to the total arm entries. To study spatial memory, we used the novel arm discrimination task, based on rodents’ innate preference to explore a novel environment more than a familiar one, with modifications [72]. We blocked one arm and allowed the mice to explore the other two arms for 5 min. After 4 h, the animals were placed again in the maze with all three arms opened and allowed to explore the familiar arms and the novel arm for another 5 min. The time spent and the number of entries in the specific area were record and analyzed.

### Open field test (OFT)

The open field test was used to assess both exploratory behavior and locomotor activity. Mice were placed in an open field (45 × 45 × 45 cm) for 10 min. Monitoring was performed with an automated tracking system (CleverSys TopScanLite). Time spent, distance in the center, and the total distance of movement were measured.

### Novel location recognition (NLR) test

The NLR test, which primarily evaluates hippocampal-dependent spatial learning, was performed as described previously [73]. Briefly, mice were placed in a cubic arena (45 × 45 × 45 cm^3^) with two identical objects, which they were allowed to explore for 10 min (training phase). One day later, animals were exposed again to two identical objects; one object was moved to a new location, at the same distance from the walls and not the release corner. Mice were allowed to explore for 10 min. Exploration time for each of the two objects was quantified. A discrimination index, defined as Time with Novel Location Object/(Time with Novel Location Object + Time with Familiar Object), was then calculated to represent the relative exploration of the novel location object.

### Immunofluorescence staining

Brain slices or primary microglia were prepared and fixed with 4% paraformaldehyde (PFA). After incubated with the mouse anti-Iba1 (1:200) and anti-CD68 (1:200).

overnight at 4 °C, samples were then incubated with the Alexa Fluor 488-conjugated goat anti-rabbit secondary antibody (1:500) and Alexa Fluor 647 Donkey anti-Rat IgG H&L (1:500) for 1 h at room temperature. *Z*-stack confocal images were captured with an Olympus FV1000MPE-B confocal microscope (Japan). The number of Iba1^+^ microglia was counted double-blindly using ImageJ software (Version 2006.02.01). Iba1^+^ CD68^+^ double-positive cells per HPF in the hippocampus of mice were analyzed by Olympus FV10-ASW 4.0 Viewer software, and the 3D microglial morphology was reconstructed by the Imaris software (Bitplane, Belfast, UK, version 9.2.0).

### Statistics

Data were analyzed using GraphPad Prism 10.1 (GraphPad Software, La Jolla, CA, USA). Average fluorescence intensity was quantified using ImageJ v1.8.0 (National Institutes of Health, Bethesda, MD, USA). Data were presented as means ± standard error of the mean (SEM). All statistical analyses were performed through unpaired two-tailed Student’s *t* tests or two-way ANOVA followed by Tukey’s multiple comparisons test, with significant differences being expressed by the *p* value. Statistical significance was set as *, *p* < 0.05; **, *p* < 0.01; ***, *p* < 0.001 and ****, *p* < 0.0001; ns, not significant.

## Supplementary Information

Supplementary Material 1. Fig S1 to S9 for multiple supplementary figures. Table S1.

## Data Availability

No datasets were generated or analysed during the current study.
